# Efficacy and safety of radiotherapy plus anti-PD1 versus transcatheter arterial chemoembolization plus sorafenib for advanced hepatocellular carcinoma: a real-world study

**DOI:** 10.1186/s13014-022-02075-6

**Published:** 2022-06-11

**Authors:** Jian-Xu Li, Wen-Xiang Deng, Shi-Ting Huang, Xiao-Feng Lin, Mei-Ying Long, Jie Zhang, Ting-Shi Su, Li-Qing Li, Ya-Dan Pang, Chun-Feng Liang, Hong-Mei Zhou, Hai-Yan Lu, Shi-Xiong Liang, Bang-De Xiang

**Affiliations:** 1 Department of Radiation Oncology, Guangxi Medical University Cancer Hospital, Nanning, 530021 China; 2 School of Public Health, Guangxi Medical University, Nanning, 530021 China; 3 Department of Hepatobiliary Surgery, Guangxi Medical University Cancer Hospital, Nanning, 530021 China

**Keywords:** Hepatocellular carcinoma, Anti-PD1, Radiotherapy, Overall survival, Transcatheter arterial chemoembolization, Sorafenib

## Abstract

**Background:**

The combination of transcatheter arterial chemoembolization (TACE) plus sorafenib prolonged progression-free survival (PFS) and overall survival (OS) than sorafenib or TACE monotherapy for patients with hepatocellular carcinoma (HCC). This study assessed the efficacy and safety of radiotherapy (RT) plus monoclonal antibody against programmed cell death 1 (anti-PD1) versus TACE plus sorafenib for patients with advanced HCC.

**Methods:**

Patients with advanced HCC who treated with RT plus anti-PD1 and TACE plus sorafenib were enrolled. Objective response rate (ORR), PFS, disease control rate (DCR) and OS were calculated to assess the antitumor response and the treatment-related adverse events to the safety.

**Results:**

Between January 2018 to March 2021, 37 patients underwent RT plus anti-PD1 and 41 patients underwent TACE plus sorafenib. The baseline characteristics between the two groups were comparable. The ORR and DCR were significantly higher in the RT + PD1 group than the TACE plus sorafenib group according to RECIST 1.1 (54.05% vs. 12.20%, *P* < 0.001; 70.27% vs. 46.37%, *P* = 0.041; respectively) and according to mRECIST (56.76% vs. 31.71%, *P* = 0.039; 70.27% vs. 46.37%, *P* = 0.041; respectively). RT plus anti-PD1 provided significantly better PFS (HR, 0.51; 95% CI 0.30–0.86; *P* = 0.017) than TACE plus sorafenib. Moreover, patients with RT plus anti-PD1 had significantly higher 3-, 6-, and 9-month OS rates than those with TACE plus sorafenib(97.3% vs. 92.30%, *P* < 0.001; 91.89% vs. 68.60%, *P* < 0.001; 75.5% vs. 60.60%, *P* < 0.001; respectively). The median OS was more favorable 17.4 months for the RT + PD1 group and 11.9 months for the TACE plus sorafenib group. No treatment-related death was observed. Grade 3 or more treatment-related adverse events (TRAEs) occurred significantly less in patients in the RT + PD1 group than the TACE plus sorafenib group (29.7% vs. 75.6%, *P* < 0.001), and all TRAEs were manageable.

**Conclusions:**

In this real-world study, RT plus anti-PD1 showed significantly promising efficacy and manageable safety than TACE plus sorafenib in patients with advanced HCC. Toxicities were manageable, with no unexpected safety signals. The study provides evidence on a new therapeutic method in the treatment of advanced HCC.

## Background

Hepatocellular carcinoma (HCC) is the most common type of liver cancer, which is the 6th most common cancer worldwide and the 3rd leading cause of cancer-related death [1]. Most patients are found at an advanced stage having a poor prognosis [2, 3]. TACE, RT and sorafenib are recommended as standard treatments for the patients with advanced HCC by The National Comprehensive Cancer Network (NCCN) guideline [4]. The combination of TACE with sorafenib is proved to be a well-tolerated and feasible treatment for patients with HCC [5, 6]. The combination of TACE and sorafenib prolonged progression-free and overall survival times than sorafenib or TACE monotherapy, and was indicated to be a promising treatment in patients with unresectable HCC [7–9].

With the rapid improvement of RT technology and equipment, the curative effect of RT for HCC has been significantly improved in recent years. Hypofractionated RT has also been shown to be effective in patients with HCC through excellent local control, downstaging, conversion from unresectable to resectable status, and treatments of unresectable HCC with vessel invasion or multiple intrahepatic metastases [10–12].

Recently, the IMbrave trial showed atezolizumab combined with bevacizumab achieved better OS and PFS than sorafenib, but the combination is expensive and the rate of grade 3 or more TRAEs was high with a high risk of grade 5 events [13]. Thus, new therapeutic strategies for advanced HCC are necessarily needed. The anti-PD1 has emerged to play a promising role in the treatment of HCC over the last few years [14]. RT can undergo a so-called immunogenic death inducing in situ vaccination and render the tumor microenvironment conducive to effector T-cell 4 recruitment and function to produce a synergistic anti-tumor immunity with anti-PD1 for durable disease control [15]. Early clinical trials combining RT with anti-PD1 showed clinical activity in several cancers including non-small cell lung cancer [16], malignant pigmented tumor [17], and esophageal squamous cell carcinoma [18]. A case report including 5 cases showed the impressive tumor control in patients with advanced HCC treated by the combination of RT and anti-PD1 [19].

To the best of our knowledge, there has been rare study to directly compare the efficacy and safety of anti-PD1 plus RT versus other strategies in the treatment of advanced HCC. Therefore, we performed this real-world study to evaluate the efficacy and safety of anti-PD1 plus RT versus TACE plus sorafenib in the treatment of advanced HCC, and seek a new approach for the treatment of advanced HCC.

## Methods

### Study design and patients

This is a retrospective real-world study that was conducted at Guangxi Medical University Cancer Hospital, Nanning, China. Consecutive patients were identified via the electronic medical records from January 2018 to March 2021. Of these patients, 99 patients with advanced HCC were screened and 78 patients were included based on the following chief eligibility criteria: (a) patients with advanced HCC not fit for radical cures such as hepatic resection or local ablation and Barcelona Clinic Liver Cancer (BCLC) B or C stage, (b) diagnosed according to histopathology, the European Association for the Study of the Liver (EASL) criteria [20] or American Association for the Study of Liver Diseases (AASLD) [21], (c) received RT plus at least one cycle synchronous, or sequential anti-PD1 or TACE plus synchronous or sequential oral sorafenib, (d) had at least one measurable lesion based on Response Evaluation Criteria in Solid Tumors (RECIST) 1.1 [22] and an Eastern Cooperative Oncology Group (ECOG) performance status 0 or 1, (e) aged between 18 and 70 years, (f) had Child–Pugh class A or B liver function, (g) an observation period of ≥ 1 months. The key exclusion criteria were patients combined intrahepatic cholangiocarcinoma, with incomplete medical information or lost to follow-up, treatment duration of sorafenib less than 1 month. A total of 78 patients were enrolled in this study with 37 in the RT + PD1 group and 41 in the TACE + sorafinib group.

The study was conducted with approval from the institutional ethics committee of Guangxi Medical University Tumor Hospital. The patient’s informed consent was obtained from all participants.

### Anti-PD1 therapy

In the RT + PD1 group, anti-PD1 included pembrolizumab (Merck Sharp & Dohme Co., Inc.), camrelizumab (Jiangsu HengRui Medicine Co., Ltd.), toripalimab (Shanghai Junshi Biosciences Co., Ltd), tislelizumab (BeiGene), and sintilimab [Innovent Biologics (Suzhou) Co. Ltd.]. Dose, method of injection and duration of anti-PD1 were according to the manufacturers instructions. The anti-PD1 were recommended to be used continually every 2 or 3 weeks until disease progression, or intolerable toxicity.

### Hypofractionated intensity modulated radiotherapy

All patients treated with RT + PD1 underwent enhanced CT scan at 2.5–5 mm slice thickness for hypofractionated intensity modulated radiotherapy (hypo-IMRT) planning. Gross tumor volume (GTV) was defined as tumor focus that was visualized on contrast imaging. The clinical target volume (CTV) was defined as GTV plus a margin of 4 ~ 5 mm as described before [23]. The target volumes and organs at risk (OARs) were contour in the MIM 6.8 system (MIM, USA). Whenever conditions permitted, CT-positron emission tomography (PET-CT) fusion and CT-magnetic resonance imaging (MRI) fusion were performed. For patients with multiple metastases, 1–5 lesions were chosen for hypo-IMRT at the discretion of the radiation oncologists. The planned target volume (PTV) was defined as GTV or CTV plus asymmetrical dilation of 1 cm in craniocaudal direction and 5 mm in axial direction to set uncertainty and respiratory movement. The hypo-IMRT plans were designed using the Monaco treatment planning system (TPS) (version 5.1) or Pinnacle 3 system (Philips, Netherlands), and performed by volumetric-modulated arc therapy (VMAT) or intensity modulated radiotherapy (IMRT). The final median radiation dose delivered was 40 Gy (range, 30–60 Gy), with fraction of 4 Gy (range, 2.5–5). The most commonly used fractionations with percentage of lesions were 10 × 4 Gy; 20 × 3 Gy; 10 × 5 Gy; 10 × 3 Gy; 20 × 2.5 Gy. The RT was delivered daily over five fractions per week, using a 6 MV X-ray linear accelerator (ELEKTA Versa-HD). Cone-beam computed tomographic (CBCT) images were used to correct the positions.

### Sorafenib therapy

In the TACE + sorafenib group, patients treated with sorafenib were prescribed with two tablets of sorafenib (200 mg tablet) twice daily (800 mg/day). If unacceptable treatment-related toxicity or disease progression occurs, dose reduction and treatment termination are allowed.

### Transarterial chemoembolization

TACE was performed as described before [24]. Briefly, all patients in the TACE + sorafenib group underwent selective arteriography of the hepatic artery to locate the tumors and the percutaneous femoral artery was punctured using the Seldinger technique. The combination of local chemotherapy drug solution (cisplatin 80 mg/m^2^ or pirarubicin 60–80 mg) and drug carriers (lipiodol or ethiodized poppy seed oil 5–15 mL) was introduced into the tumor. And blank CalliSpheres R microspheres was used to embolize the feeding artery of tumors Complete embolization of the tumor supplying artery with no tumor staining observed by angiography at the end of procedure was defined as a technical success. TACE was performed 1–2 months before or after sorafenib and repeated one to six times (median, 3) at 3–6-week intervals if the patient could tolerate and consent of further treatment.

### Evaluation of efficacy and safety

The on-study date was defined as the day of acceptance of informed consent of sorafinib or hypo-IMRT planning. All TRAEs were recorded from on-study date until 30 days after last anti-PD1 injection or sorafinib, last follow-up as appropriate according to the Common Terminology Criteria for Adverse Events of the National Cancer Institute v5.0 (CTCAE 5.0). To evaluate tumour responses, RECIST 1.1 and modified Response Evaluation Criteria in Solid Tumors (mRECIST) [25] were used, respectively. The OS was defined as the on-study date to death from any cause. PFS was defined as the on-study date until disease progression or death. the ORR was defined as the proportion of patients with complete response (CR) or partial response (PR). Disease control was defined as the sum rate of CR + PR + stable disease (SD). All patients received follow-up visits every month for progression and survival status.

### Statistical analysis

The data of all patients was collected through electronic medical records and enter the data into Research Electronic Data Capture (REDCap) [26]. Through the normal distribution test by visualization on a histogram and with Kolmogorov–Smirnov tests, the continuous variables conforming to the normal distribution were expressed as the mean ± standard deviation and visualized on a histogram and with Kolmogorov–Smirnov tests. The categorical variables were expressed as number and percentage. The t-test was used for the continuous variables conforming to normal distributions, and the Pearson’s chi-square tests was used for categorical variables. OS and PFS were calculated and median OS and PFS were estimated for both groups by using Kaplan–Meier methods with the values compared using the Breslow generalized Wilcoxon test [27]. HR and CI were estimated using univariate Cox proportional risk model, and statistical analysis and forest mapping were performed using R 4.0.5 for the sub­groups. All statistical tests were two tailed. IBM SPSS software (ver. 26.0 SPSS Inc., Chicago, IL, USA) was used for the statistical analysis unless otherwise indicated.

## Results

### Patients

**Fig. 1:**
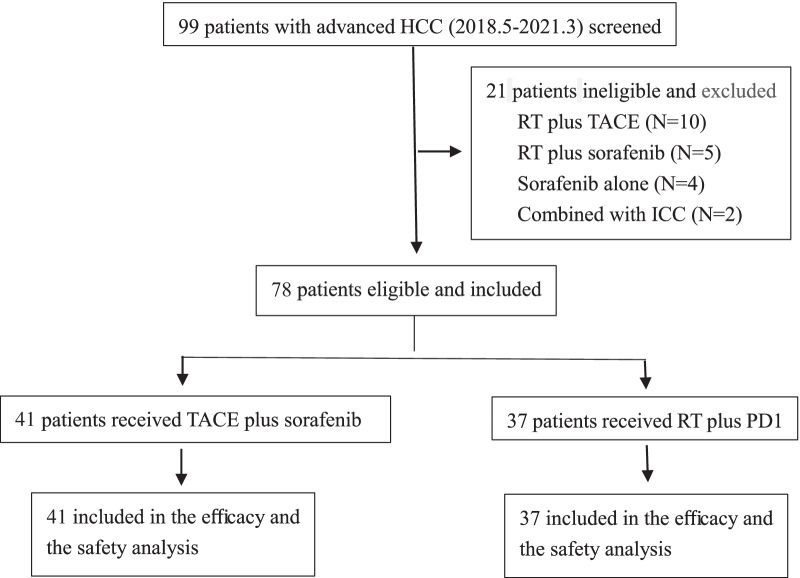
Patient selection flow. *HCC* Hepatocellular carcinoma; *TACE* Transcatheter arterial chemoembolization; *ICC* Intrahepatic cholangiocarcinoma; *RT* Radiotherapy; *PD1* Monoclonal antibody against programmed cell death

Of the 78 patients with advanced HCC enrolled, 37 (47.4%) were in the RT + PD1 group and 41 (52.6%) were in the TACE + sorafenib group (Fig. 1). In the RT + PD1 group, there were 35 patients received PD1 inhibition during RT, and 2 patients 1 months after the last RT fraction. The baseline characteristics of these patients were summarized and comparable between the two groups (Table 1). There were no significant differences in the distribution of gender, age, bodyweight, chronic hepatitis B/C virus infection, liver cirrhosis, alpha fetoprotein, albumin–bilirubin (ALBI) grade,maximum tumor diameter, macrovascular invasion, BCLC stage, hemoglobin, platelet count, white blood cell, and prior therapy between the two groups (Table 1). Patients with extrahepatic metastasis were more prevalent in the RT + PD1 group than in the TACE + sorafenib group (59.5% vs. 35.7%, respectively, *P* = 0.044), while Albumin (< 35 g/L) were more common in the TACE plus sorafenib group than in the TACE plus RT group (76.2% vs. 46.0%, respectively; *P* = 0.010).

**Table 1:** Patient baseline demographic and clinical characteristics

Variables	RT + PD1, *N* = 37 (%)	TACE + Sorafenib, *N* = 41(%)	*P* value
			RT + PD1 versus TACE + sorafenib
Gender, male	35(94.60%)	37(90.24%)	0.678
Age, year	54.16 ± 10.68	51.71 ± 9.571	0.288
Bodyweight,kg	63.58 ± 9.96	65.06 ± 10.84	0.534
Hepatitis B, present	31(83.78%)	30(73.17%)	0.286
Hepatitis C, present	1(2.70%)	5(12.2%)	0.204
Liver cirrhosis, present	17(45.95%)	26(63.41%)	0.171
Alpha fetoprotein, ≥ 400 ng/ml	18(48.65%)	18(43.90%)	0.820
ALBI grade, ≤ 2	36 (97.3%)	38 (93.7%)	0.617
Blood bilirubin, > 21umol/L	9(24.32%)	14(34.15%)	0.457
Albumin, < 35 g/L	17(45.95%)	31(75.61%)	0.010
Hemoglobin, < 131 g/L	22(59.46%)	19(46.34%)	0.266
Platelet count, < 100*10^9/^L	7(18.92%)	6(14.63%)	0.763
White blood cell, < 3.97*10^9/^L	7(18.92%)	4(9.76%)	0.333
Maximum tumor diameter, cm	7.30(4.75–8.65)	6.20(4.15–10.80)	0.860
Maximum tumor diameter, ≥ 10 cm	8(21.62%)	13(31.71%)	0.444
Macrovascular invasion, present	16(43.24%)	24(58.54%)	0.257
Extrahepatic metastasis,present	22(59.46%)	14(34.15%)	0.040
BCLC stage			0.147
B	4(10.81%)	10(24.39%)	
C	33(89.19%)	31(75.61%)	
Prior therapy			
TACE, present	11(29.73%)	10(24.39%)	0.619
Hepatectomy, present	16(43.24%)	14(34.15%)	0.487
Systemic therapy, present	6(16.22%)	6(14.63%)	1.000

Data are mean ± standard deviation, median (IQR) or *N* (%)

*RT* Radiotherapy; *PD1* The monoclonal antibody against programmed cell death 1; *TACE* Transcatheter arterial chemoembolization; *ALBI* Albumin–bilirubin; *BCLC* Barcelona Clinic Liver Cancer

In the RT + PD1 group, patients received median 5 (1–20) cycles anti-pd1 including 32 patients with camrelizumab (SHR-1210, Jiangsu HengRui Medicine Co., Ltd.) [28], 3 with sintilimab [IBI308, Innovent Biologics (Suzhou) Co. Ltd.] [29], 2 with tislelizumab (BGB-A317, BeiGene) [30]. All patients with much more lesions than hypo-IMRT targets received hypo-IMRT median dose 40 Gy (rang 30–60 Gy) with median 4 Gy per fractions (rang 2.5–5 Gy per fractions). The median duration of sorafinib therapy was 6.5 (range 1–20) months and the median number of TACE sessions was 2 (range 1–6) in the TACE + sorafenib group.

At data cutoff, 14 (37.8%) patients had received subsequent therapy including 1 received TACE plus hepatectomy, 2 received regorafenib, 2 received sorafinib, 2 received lenvatinib,3 received TACE, and 4 received apatinib in the RT + PD1 group after disease progression. As to the TACE + sorafenib group, 16 (39.0%) patients had received subsequent therapy including one received radiofrequency ablation plus anti-PD1, 1 received hepatectomy plus anti-PD1, 1 received lenvatinib, 1 received lenvatinib plus anti-PD1, 1 received apatinib, 1 received regorafenib plus anti-PD1, 2 received hepatectomy, and 10 received anti-PD1.

### Radiologic response after treatment

**Fig. 2:**
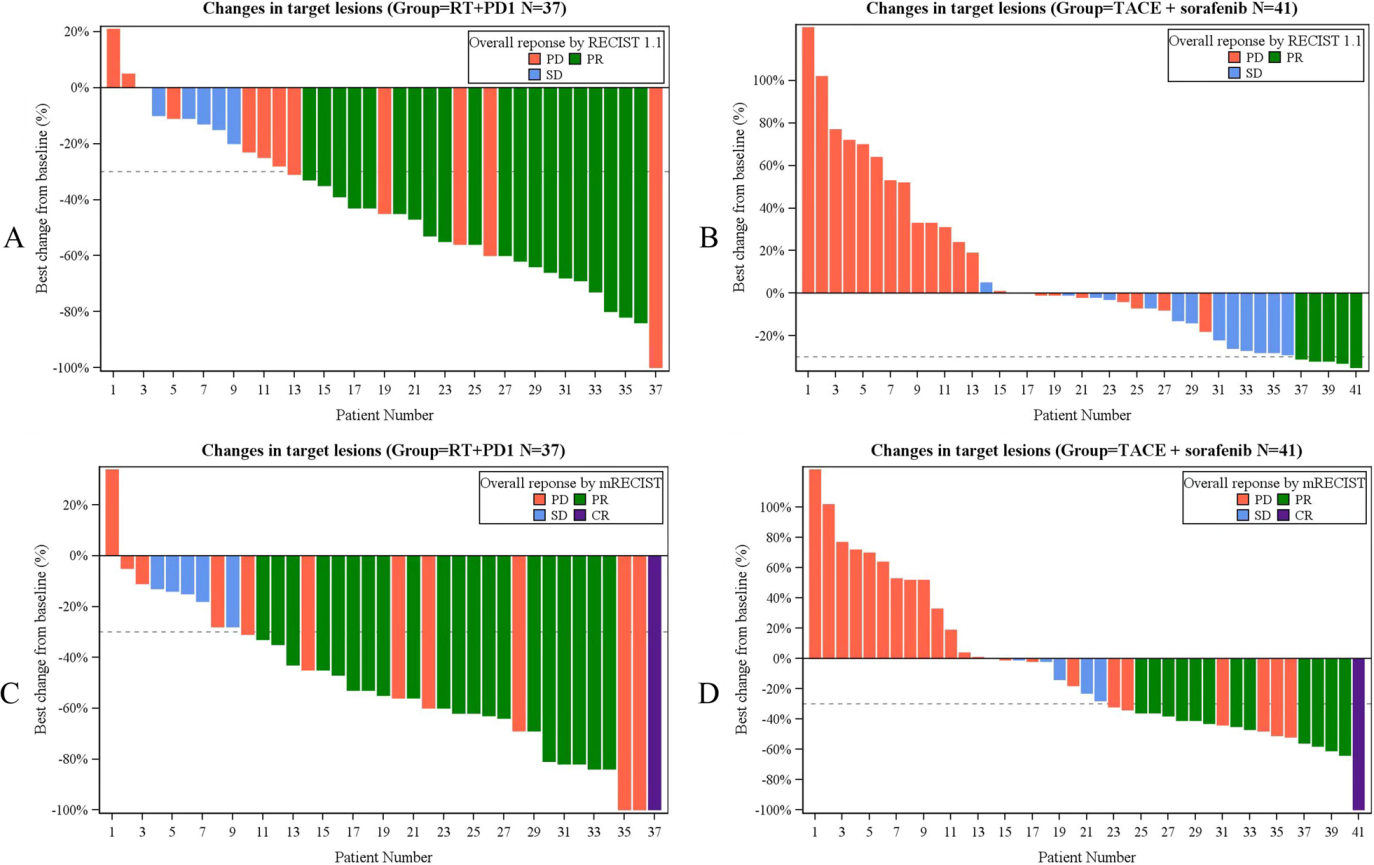
Best percentage change from baseline in sums of diameters of target lesions. **A** In the RT + PD1 group based on RECIST 1.1, **B** In the TACE plus sorafenib group based on RECIST 1.1, **C** In the RT + PD1 group based on mRECIST, **D** In the TACE plus sorafenib group based on mRECIST. *RT* Radiotherapy; *PD1* Monoclonal antibody against programmed cell death; *TACE* Transcatheter arterial chemoembolization; *RECIST 1.1* Response Evaluation Criteria in Solid Tumors 1.1; *mRECIST* Modified Response Evaluation Criteria in Solid Tumors; *CR* Complete response; *PR* Partial response; *SD* Stable disease; *PD* Progressive disease

The median follow-up of the patients in the RT + PD1 group was 13.4 months (95% CI 12.1–14.6), and in the TACE plus sorafenib group was 16.6 months (95% CI 14.9–18.3), *P* = 0.034. The tumor responses are presented in Table 2. the PR rate, ORR and DCR were significantly higher in the RT + PD1 group than the TACE plus sorafenib group according to RECIST 1.1 (54.05% vs. 12.20%, *P* < 0.001; 54.05% vs. 12.20%, *P* < 0.001; 70.27% vs. 46.34%, *P* = 0.041; respectively) and according to mRECIST (56.76% vs. 31.71%, *P* = 0.039; 54.05% vs. 12.20%, *P* < 0.001; 70.27% vs. 46.34%, *P* = 0.041; respectively). Best percent change from baseline in sum of longest diameters for target lesions per patient in the two groups is shown in the waterfall plot (Fig. 2), where Fig. A, B, C, and D represent the results in the RT + PD1 group based on RECIST 1.1, the TACE plus sorafenib group based on RECIST 1.1, the RT + PD1 group based on mRECIST, and the TACE plus sorafenib group based on mRECIST, respectively.

**Table 2:** Summary of efficacy outcomes

Variables	RT + PD1, *N* = 37 (%)	TACE + Sorafenib, *N* = 41(%)	*P* value
*According to RECIST 1.1*			
Confirmed objective response	20 (54.05%)	5(12.20%)	< 0.001
Time to response, months, (IQR)	1.80(1.57–2.00)	2.87(1.407–6.86)	0.467
Disease control	26(70.27%)	19(46.34%)	0.041
*Best overall response*			
CR	0(0)	0(0)	1.000
PR	20 (54.05%)	5(12.20%)	< 0.001
SD	6(16.22%)	14(34.15%)	0.118
PD	11(29.73%)	22(53.66%)	0.041
Progression-free survival, months, median, (95% CI)	5.86(3.19–8.53)	3.70(2.60–4.80)	0.017
*According to mRECIST*			
Confirmed objective response	21(56.76%)	13(31.71%)	0.039
Time to response, months, (IQR)	1.85(1.60–2.98)	1.47(0.80–2.60)	0.061
Disease control	26(70.27%)	19(46.34%)	0.041
*Best overall response*			
CR	1(2.70%)	1(2.44%)	1.000
PR	20 (54.05%)	12(29.27%)	0.038
SD	5(13.51%)	6(14.63%)	0.774
PD	11(29.73%)	22(53.66%)	0.041
Progression-free survival, months, median, (95% CI)	5.86(3.19–8.53)	3.70(2.60–4.80)	0.019
*Overall survival*			
3-month, %	97.30%	92.30%	< 0.001
6-month, %	91.90%	68.60%	< 0.001
9-month, %	75.50%	60.60%	< 0.001
12-month, %	52.20%	47.5%	0.061
Overall survival, months, median, (95% CI)	17.40 (8.69–26.11)	11.90 (6.35–17.45)	0.146

Data are *N* (%; 95% CI), unless indicated

*RT* Radiotherapy; *PD1* The monoclonal antibody against programmed cell death 1; *TACE* Transcatheter arterial chemoembolization; *RECIST 1.1* Response Evaluation Criteria in Solid Tumors 1.1; *mRECIST* Modified Response Evaluation Criteria in Solid Tumors; *CR* Complete response; *PR* Partial response; *SD* Stable disease; *PD* Progressive disease

### Progression-free survival analysis

**Fig. 3:**
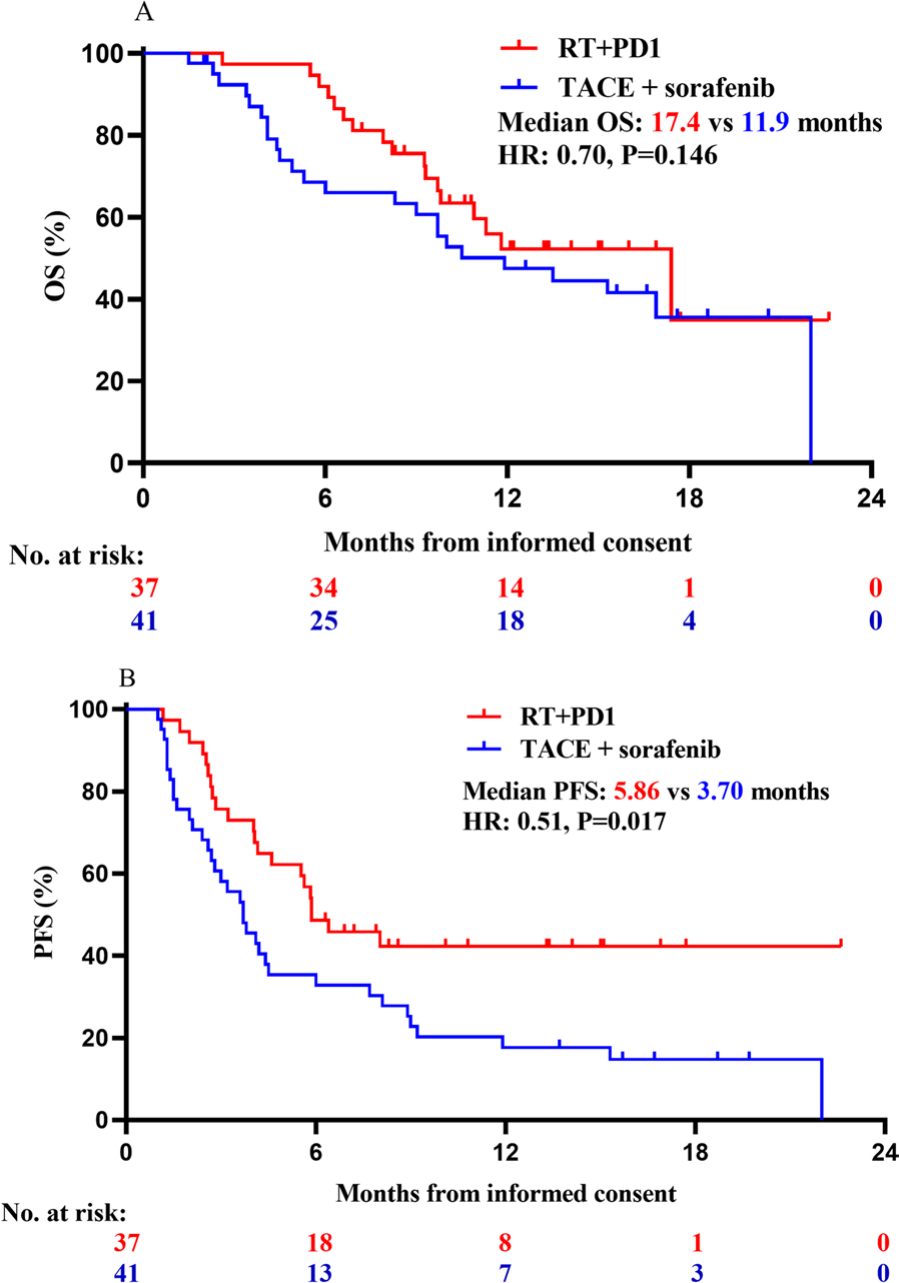
Kaplan–Meier analysis of overall and progression free survival. **A** PFS and **B** OS for all patients. *HR* Hazard ratio; *RT* Radiotherapy; *PD1* Monoclonal antibody against programmed cell death; *TACE* Transcatheter arterial chemoembolization; *OS* Overall survival; *PFS* Progression-free survival

**Fig. 4:**
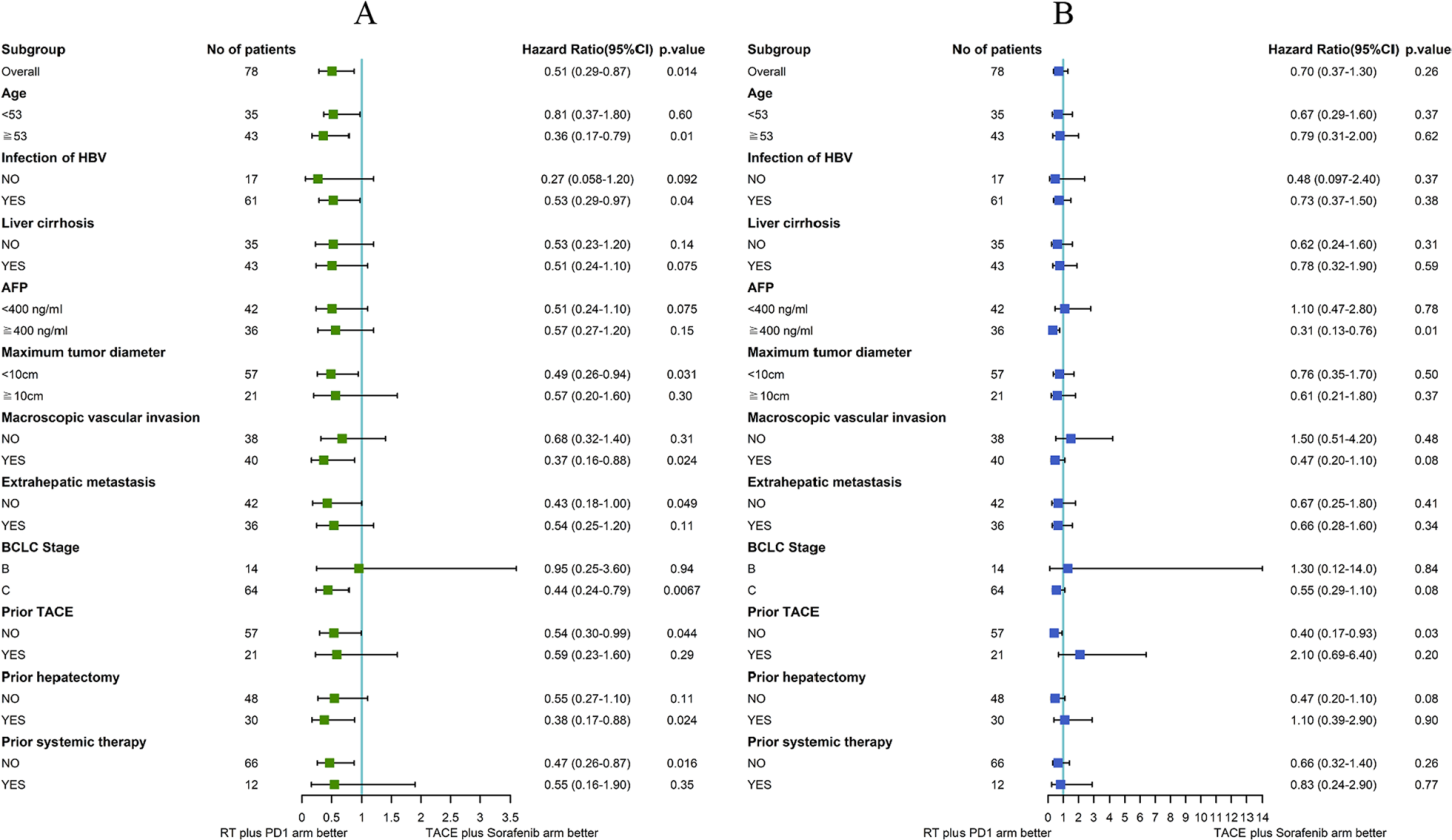
Forest plot of PFS **A** and OS **B** in subgroups of patients. *RT* Radiotherapy; *PD1* Monoclonal antibody against programmed cell death; *TACE* Transcatheter arterial chemoembolization; *OS* Overall survival; *PFS* Progression-free survival; *HBV* Hepatitis B; *BCLC* Barcelona Clinic Liver Cancer; *AFP* Alpha-fetoprotein

Progression of the disease was observed in 37 of the patients (47.4%) who underwent RT + PD1 and 41 of the patients who underwent TACE plus sorafenib group (52.8%) during follow-up. Patients in the PD1 + RT group had significant better PFS compared with those in the TACE + sorafenib group (5.86 vs. 3.70 months; HR, 0.51; 95% CI 0.30–0.86; *P* = 0.017; Table 2 and Fig. 3). The median PFS in the PD1 + RT group had significant longer than TACE + sorafenib group across sub­groups based on age ≥ 53, infection of hepatitis B (HBV), the maximum tumor diameter < 10 cm, MVI, without extrahepatic metastasis, BCLC stage C, no prior TACE, prior hepatectomy, and no prior systemic therapy (*P* = 0.01, 0.04, 0.031, 0.024, 0.049, 0.0067, 0.044, 0.024 and 0.016; respectively, Fig. 4A). The effect of the two groups on median PFS was consistent across sub­groups based on other baseline characteristics (Fig. 4A).

### Overall survival analysis

Among the 37 patients in the RT + PD1 group, 18 are still alive, 18 have died, and 1 is lost to follow-up by the end of this study. Among the 41 patients who received TACE plus sorafenib, 13 are still alive, 25 have died, and 3 are lost to follow-up. The 3-, 6-, and 9-month OS rates were significant better in the RT + PD1 group than that in the TACE plus sorafenib (97.3% vs. 92.30%, *P* < 0.001; 91.89% vs. 68.60%, *P* < 0.001; 75.5% vs. 60.60%, *P* < 0.001; respectively, Table 2). The median OS was 17.4 months for the RT + PD1 group and 11.9 months for the TACE plus sorafenib group (HR: 0.70, 95% CI, 0.38–1.30, *P* = 0.146; Table 2 and Fig. 3B). There were no significant differences for the median OS in the distribution of the two groups, but a trend toward a more favorable outcome in the RT + PD1 group.

**Fig. 5:**
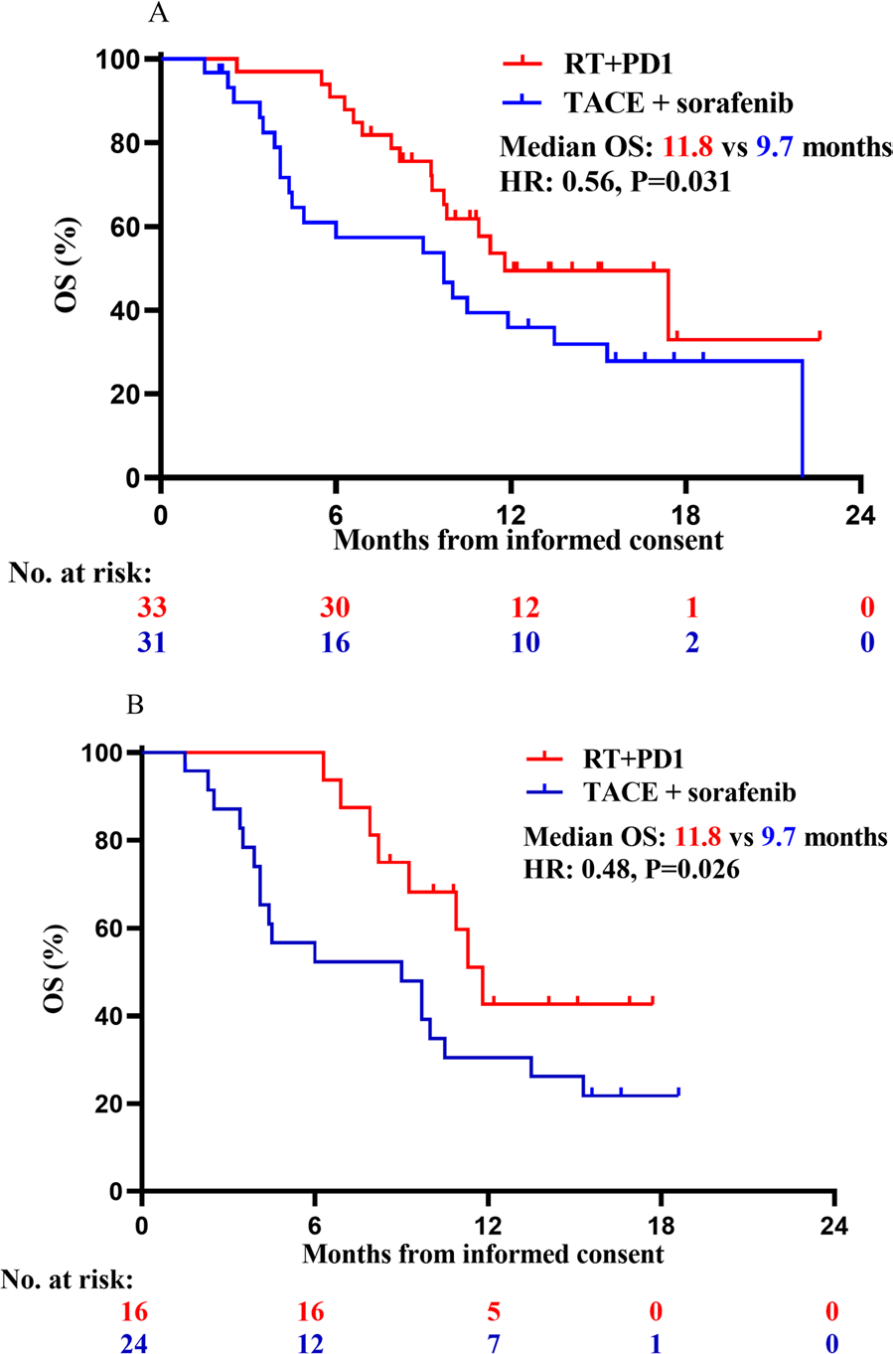
Kaplan–Meier analysis of overall and progression free survival in patient subgroups. **A** OS among patients with BCLC-C; **B** OS among patients with MVI; *RT* Radiotherapy; *PD1* Monoclonal antibody against programmed cell death; *TACE* Transcatheter arterial chemoembolization; *OS* Overall survival; *PFS* Progression-free survival; *BCLC-C* Barcelona Clinic Liver Cancer C stage; *MVI* Macrovascular invasion; *AFP* Alpha-fetoprotein

Patients in the RT + PD1 group had statistically significant better OS than those in the TACE plus sorafenib group with AFP level ≥ 400 ng/ml, BCLC stage C and MVI (11.8 vs. 9.7 months, *P* = 0.026; 11.8 vs. 9.7 months, *P* = 0.031; 11.8 vs. 9.7 months, *P* = 0.026; respectively, Fig. 5). Although, there had not significant better OS with BCLC stage C and MVI using univariate Cox proportional risk model and R 4.0.5. The effect of the two groups on median OS was consistent across sub­groups based on other baseline characteristics (Fig. 4B).

### Safety profile

The TRAEs, which occurred in ≥ 10% of patients, are summarized in Table 3. No deaths were attributed to TRAEs in this study. The TRAEs in the RT + PD1 group show less than in the TACE plus sorafenib group, including increased AST (54.05% vs 92.68%, *P* < 0.001), increased AST (51.35% vs. 80.49%, *P* = 0.008), hand-foot skin reaction (HFSR) (0% vs. 53.66%, *P* < 0.001). Decreased hemoglobin was more frequent in the RT + PD1 group than in the TACE plus sorafenib group (89.19% vs. 68.29%, *P* = 0.031). There were no differences in regard to other TRAEs such as decreased albumin, decreased white blood cell, increased blood bilirubin and decreased platelet count. Grade 3 or more TRAEs occurred significantly less in patients in the RT + PD1 group than the TACE plus sorafenib group (29.7% vs. 75.6%, *P* < 0.001). The major grade 3 or more TRAEs were observed significantly milder in the RT + PD1 group than in the TACE plus sorafenib group, including increased aspartate aminotransferase (AST) (5.41% vs. 58.54%, *P* < 0.001), increased alanine aminotransferase (ALT) (5.41% vs. 36.59%, *P* < 0.001) and hand-foot skin reaction (0% vs. 14.63%, *P* = 0.027). The two groups did not differ in terms of other grade 3 or more TRAEs including decreased hemoglobin, decreased albumin, decreased white blood cell, increased blood bilirubin and decreased platelet count.

**Table 3:** Treatment-related adverse events based on CTCAE 5.0

Adverse events	RT + PD1, *N* = 37 (%)		TACE + sorafenib, *N* = 41(%)		*P* value	
	Any grade (%)	Grade 3–4 (%)	Any grade (%)	Grade 3–4 (%)	Any grade	Grade 3–4
Increased AST	20(54.05)	2(5.41)	38(92.68)	24(58.54)	< 0.001	< 0.001
Increased ALT	19(51.35)	2(5.41)	33(80.49)	15(36.59)	0.008	0.001
Hand-foot skin reaction	0(0)	0(0)	22(53.66)	6(14.63)	< 0.001	0.027
Decreased hemoglobin	33(89.19)	5(13.51)	28(68.29)	2(4.88)	0.031	0.247
Decreased albumin	30(81.08)	0(0)	37(90.24)	1(2.44)	0.333	1.000
Decreased white blood cell	28(75.68)	3(8.11)	12(29.27)	1(2.44)	< 0.001	0.341
Increased blood bilirubin	20(54.05)	1(2.70)	16(39.02)	1(2.44)	0.256	1.000
Decreased platelet count	19(51.35)	8(21.62)	14(34.115)	5(12.20)	0.169	0.364

Data are *N* (%)

*CTCAE 5.0* The Common Terminology Criteria for Adverse Events of the National Cancer Institute v5.0; *RT* Radiotherapy; *PD1* The monoclonal antibody against programmed cell death 1; *TACE* Transcatheter arterial chemoembolization; *AST* Aspartate aminotransferase; *ALT* Alanine aminotransferase

## Discussion

Few studies have compared the efficacy and safety of RT + anti-PD1 therapy vs. TACE + sorafenib for treating advanced HCC. In the present real-world study, RT + anti-PD1 therapy was associated with significantly better PFS and ORR, as well as milder, more acceptable TRAEs. Thus, combining anti-PD1 therapy with RT may be an effective, safe treatment for advanced HCC.

Sorafenib is recommended as the first-line treatment for the patients with advance HCC [4], in whom the drug was associated with median OS of 6.5 months, DCR of 35.3%, and ORR of 3.3% in a study of patients in the Asia–Pacific region [31], or 10.4 months, 64%, and 4% in the ORIENT-32 study [29]. We found that combining sorafenib with TACE led to better outcomes, as did using RT + anti-PD1 therapy. Similarly, TACE + sorafenib has been associated with better outcomes than TACE or sorafenib alone in patients with unresectable HCC [6, 8, 9, 32]. In the GIDEON study of patients treated with sorafinib, median OS was 12.7 months among those previously treated with TACE group, 9.2 months among those not previously treated with TACE. The median OS of our patients treated by TACE + sorafenib was 11.9 months, similar to the patients previously treated by TACE in the GIDEON study [6]. This similarity, despite the much higher proportions of our patients in BCLC stages A or B or with chronic HBV infection, strongly suggests that combining TACE and sorafenib can benefit patients with advanced HCC.

In our real-word study, the PR rate, ORR and DCR were significantly higher in the RT + PD1 group than the TACE + sorafenib group, according to both RECIST 1.1 and mRECIST. In fact, ORR was better in our study (54.05%) than in the IMbrave150 trial (27.3%) [13] or the REFLECT trial (24.1%) [33]. We observed significantly better PFS in patients treated with RT + anti-PD1 than in those treated with TACE + sorafenib. OS at 3, 6, or 9 months was significantly better in the RT + PD1 group than in the TACE + sorafenib group, and the 6-month OS in our RT + PD1 group (91.89%) was better than that in the IMbrave150 trial (84.8%) [13]. Moreover, there was a trend toward longer median OS in the RT + PD1 group than in the TACE + sorafenib group (17.4 months vs. 11.9 months), and the failure of this difference to achieve significance may reflect the significantly shorter median follow-up in the RT + PD1 group (13.4 months vs 16.6 months). These findings suggest that RT + anti-PD1 and TACE + sorafenib can benefit patients with advanced HCC.

Subgroup analysis revealed that the median PFS in the PD1 + RT group was significantly longer than in the TACE + sorafenib group across subgroups based on age ≥ 53, HBV infection, maximum tumor diameter < 10 cm, MVI, absence of extrahepatic metastasis, BCLC stage C, no prior TACE, prior hepatectomy, or no prior systemic therapy. RT + PD1 was associated with significantly longer OS than TACE + sorafenib among patients with an AFP level ≥ 400 ng/ml, BCLC stage C and MVI. However, the OS difference did not achieve significance among patients with BCLC stage C or MVI, which may be explained by the shorter follow-up time for the PD1 + RT group. Together, these findings may help select patients with advanced HCC who may particularly benefit from combining RT and anti-PD1 or combining TACE and sorafenib therapy.

The spectrum, incidence, and severity of adverse events in the TACE + sorafenib group were consistent with safety profiles in other studies [5, 7–9, 34]. There were fewer total TRAEs and fewer grade 3 or above TRAEs in the RT + PD1 group than in the TACE + sorafenib group, including increased AST, AST and hand-foot skin reaction.

This study had several limitations. Its retrospective design was the main drawback, which may increase the risk of confounding due to differences in adherence or socioeconomic status. Second, the follow-up period was significantly shorter in the RT + PD1 group than in the TACE + sorafenib group, yet it was long enough to evaluate PFS and tumor response, which may be better indicators of efficacy than OS [35]. Third, three types of anti-PD1 drugs were used in this study and they may differ in efficacy, which may have confounded our results. Fourth, we were unable to assess some adverse events such as cardiotoxicity for lack of standard hematological test data.

## Conclusion

Among patients with advanced HCC, RT + anti-PD1 therapy may be associated with significantly better PFS, PR rate, ORR and DCR than TACE + sorafenib, as well as better OS among subgroups of patients in BCLC stage C, or with macrovascular invasion or AFP ≥ 400 ng/ml. Adverse events were milder in patients treated with RT + anti-PD1 therapy than in those treated with TACE + sorafenib. Our results, which should be verified in larger studies with longer follow-up, support RT + anti-PD1 therapy as a potential treatment for advanced HCC.

## Data Availability

The data underlying this article will be shared on reasonable request to the corresponding author.

## Abbreviations

TACE: Transcatheter arterial chemoembolization
PFS: Progression-free survival
OS: Overall survival
HCC: Hepatocellular carcinoma
RT: Radiotherapy
anti-PD1: Monoclonal antibody against programmed cell death 1
ORR: Objective response rate
DCR: Disease control rate
HR: Hazard ratio
TRAE: Treatment-related adverse events
NCCN: The national comprehensive cancer network
BCLC: Barcelona clinic liver cancer
EASL: European association for the study of the liver
AASLD: American association for the study of liver diseases
RECIST: Response evaluation criteria in solid tumors
ECOG: Eastern cooperative oncology group
hypo-IMRT: Hypofractionated intensity modulated radiotherapy
CTCAE 5.0: Common terminology criteria for adverse events of the national cancer institute v5.0
mRECIST: Modified response evaluation criteria in solid tumors
CR: Complete response
PR: Partial response
SD: Stable disease
ALBI: Albumin–bilirubin
MVI: Macrovascular invasion
HBV: Chronic hepatitis B virus infection
AFP: Alpha-fetoprotein
ALT: Alanine aminotransferase
AST: Aspartate aminotransferase
